# Supplementary material for: Temporal Trends and Differences in Sexuality among Depressed and Non-Depressed Adults in the United States
Source: Int J Environ Res Public Health. 2022 Oct 27;19(21):14010. doi: 10.3390/ijerph192114010 (PMC9654390; doi:10.3390/ijerph192114010)
Supplement: Supplementary file 1 [file ijerph-19-14010-s001.zip › ijerph-1969813-SI.pdf]

TableS1 Differences between Depressed and Non-depressed in 2005-2008

**2005-2008**	**Depressed (N=1742)**	**Non-Depressed (N=5322)**	**Depressed VS. Non-Depressed**
**Age (Y)**	38.66(37.94-39.38)	38.45(37.95-38.95)	
**Female(%)**	59.99	48.95	
**Age group(%)**			
18-38(Y)	46.17(42.87-49.46)	49.77(47.85-51.69)	
39-59(Y)	53.83(50.54-57.13)	50.23(48.31-52.15)	
**Race/Ethnicity (%)**			
Non-Hispanic White	65.30(59.99-70.62)	68.99(64.35-73.63)	
Non-Hispanic Black	13.19(10.01-16.36)	11.67(8.71-14.63)	
Hispanic	15.62(11.96-19.27)	13.67(11.13-16.21)	
Others	5.89(4.41-7.38)	5.67(4.41-6.93)	
**Marital Status (%)**			
Living Together	55.60(51.22-59.97)	65.39(62.92-67.87)	
Living Alone	42.05(37.78-46.32)	32.28(29.80-34.75)	
**Employment Status (%)**			
Employed	63.14(59.26-67.02)	81.47(79.82-83.12)	
Unemployed	36.86(32.98-40.74)	18.52(16.87-20.17)	
**Education Status (%)**			
Some college or Above	47.75(43.77-51.73)	59.63(56.28-62.98)	
High school graduate or GED	25.75(22.89-28.60)	21.89(20.14-23.64)	
Below High School	22.08(18.82-25.35)	13.87(11.79-15.95)	
**Family Income Status (%)**			
PIR <1.30	28.30(24.83-31.78)	15.79(13.94-17.65)	
PIR: 1.30-3.50	33.82(30.42-37.22)	30.99(28.53-33.45)	
PIR ≥3.50	32.57(28.37-36.77)	49.05(45.41-52.69)	
**Average Age of First Sexual Intercourse (Y)**	16.65(16.31-16.98)	17.59(17.35-17.82)	-0.94(-1.30--0.58)
**Normal Frequency of Sexual Activity (%)**	24.98(22.49-27.48)	28.73(26.96-30.50)	-3.75(-6.77--0.73)
**Heterosexual Sexual Orientation (%)**	62.88(59.77-65.98)	70.89(69.01-72.77)	-8.01(-11.60--4.43)

GED: General Educational Development; PIR, Poverty Impact Ratio. Values are mean for continuous variables and percentage for categorical variables; Values in the brackets represent the confidence interval of the corresponding variables.

TableS2 Differences between Depressed and Non-depressed in 2009-2012

2009-2012	Depressed (N=1921)	Non-Depressed (N=5253)	Depressed VS. Non-Depressed
**Age (Y)**	39.01(38.41-39.61)	38.54(37.67-39.40)	
**Female(%)**	58.3	46.49	
**Age group(%)**			
18-38(Y)	46.36(43.79-48.93)	48.86(45.55-52.18)	
39-59(Y)	53.64(51.07-56.21)	51.14(47.82-54.45)	
**Race/Ethnicity (%)**			
Non-Hispanic White	62.76(56.39-69.12)	65.06(59.64-70.48)	
Non-Hispanic Black	13.77(9.95-17.59)	11.47(9.11-13.84)	
Hispanic	16.84(12.30-21.38)	15.67(11.52-19.82)	
Others	6.63(4.89-8.38)	7.79(6.22-9.37)	
**Marital Status (%)**			
Living Together	49.90(47.45-52.34)	61.60(58.60-64.59)	
Living Alone	45.03(42.08-47.98)	33.87(31.34-36.40)	
**Employment Status (%)**			
Employed	57.96(54.50-61.41)	76.45(74.44-78.45)	
Unemployed	42.04(38.59-45.50)	23.52(21.53-25.52)	
**Education Status (%)**			
Some college or Above	51.55(46.47-56.62)	62.89(59.92-65.86)	
High school graduate or GED	23.34(19.89-26.79)	19.10(17.37-20.84)	
Below High School	20.16(17.63-22.70)	13.40(11.13-15.67)	
**Family Income Status (%)**			
PIR <1.30	34.23(30.19-38.28)	19.54(16.74-22.33)	
PIR: 1.30-3.50	34.76(31.57-37.95)	31.17(28.40-33.94)	
PIR ≥3.50	24.94(21.38-28.51)	43.17(39.50-46.85)	
**Average Age of First Sexual Intercourse (Y)**	16.42(16.15-16.69)	17.39(17.20-17.58)	-0.97(-1.30--0.64)
**Normal Frequency of Sexual Activity (%)**	25.07(22.36-27.77)	31.50(29.69-33.30)	-6.43(-9.64--3.22)
**Heterosexual Sexual Orientation (%)**	66.69(64.07-69.32)	78.28(76.33-80.22)	-11.59(-14.81--8.36)

GED: General Educational Development; PIR, Poverty Impact Ratio. Values are mean for continuous variables and percentage for categorical variables; Values in the brackets represent the confidence interval of the corresponding variables.

TableS3 Differences between Depressed and Non-depressed in 2013-2016

2013-2016	Depressed (N=1769)	Non-Depressed (N=5430)	Depressed VS. Non-Depressed
**Age (Y)**	39.45(38.48-51.20)	38.34(37.80-38.87)	
**Female(%)**	61.67	48.36	
**Age group(%)**			
18-38(Y)	47.31(43.43-51.20)	50.50(48.16-52.83)	
39-59(Y)	52.69(48.80-56.57)	49.50(47.17-51.84)	
**Race/Ethnicity (%)**			
Non-Hispanic White	61.16(55.29-67.03)	61.52(55.85-67.19)	
Non-Hispanic Black	13.11(9.70-16.53)	11.59(8.78-14.39)	
Hispanic	16.78(13.49-20.07)	17.89(13.55-22.22)	
Others	8.95(6.86-11.03)	9.00(7.51-10.50)	
**Marital Status (%)**			
Living Together	50.69(47.43-53.95)	64.24(62.01-66.46)	
Living Alone	45.19(42.10-48.28)	31.49(29.29-33.69)	
**Employment Status (%)**			
Employed	61.31(57.37-65.26)	79.59(77.52-81.66)	
Unemployed	38.66(34.71-42.61)	20.30(18.22-22.38)	
**Education Status (%)**			
Some college or Above	56.38(52.58-60.18)	65.01(61.43-68.60)	
High school graduate or GED	22.59(19.28-25.89)	18.75(16.75-20.75)	
Below High School	16.98(14.25-19.71)	11.97(9.75-14.20)	
**Family Income Status (%)**			
PIR <1.30	32.05(28.09-36.00)	19.64(16.56-22.73)	
PIR: 1.30-3.50	35.11(31.11-39.11)	31.82(29.86-33.79)	
PIR ≥3.50	26.78(23.27-30.29)	42.60(38.40-46.80)	
**Average Age of First Sexual Intercourse (Y)**	16.46(16.23-16.69)	17.43(17.22-17.63)	-0.97(-1.22--0.72)
**Normal Frequency of Sexual Activity (%)**	26.38(24.27-28.50)	30.46(28.17-32.74)	-4.07(-7.15--1.00)
**Heterosexual Sexual Orientation (%)**	68.48(64.51-72.45)	77.49(76.05-78.94)	-9.01(-13.18--4.84)

GED: General Educational Development; PIR, Poverty Impact Ratio. Values are mean for continuous variables and percentage for categorical variables; Values in the brackets represent the confidence interval of the corresponding variables.
